# Deficiency syndromes in top predators associated with large-scale changes in the Baltic Sea ecosystem

**DOI:** 10.1371/journal.pone.0227714

**Published:** 2020-01-09

**Authors:** Sanna Majaneva, Emil Fridolfsson, Michele Casini, Catherine Legrand, Elin Lindehoff, Piotr Margonski, Markus Majaneva, Jonas Nilsson, Gunta Rubene, Norbert Wasmund, Samuel Hylander

**Affiliations:** 1 Department of Biology and Environmental Sciences, Centre for Ecology and Evolution in Microbial model Systems–EEMiS, Linnaeus University, Kalmar, Sweden; 2 Department of Arctic and Marine Biology, UiT The Arctic University of Norway, Tromsø, Norway; 3 Department of Biology, Norwegian University of Science and Technology, Trondheim, Norway; 4 Swedish University of Agricultural Sciences, Department of Aquatic Resources, Institute of Marine Research, Lysekil, Sweden; 5 National Marine Fisheries Research Institute, Gdynia, Poland; 6 NTNU University Museum, Norwegian University of Science and Technology, Trondheim, Norway; 7 Fish Resources Research Department, Institute of Food Safety, Animal Health and Environment BIOR, Riga, Latvia; 8 Leibniz-Institute for Baltic Sea Research, Warnemünde, Germany; National Taiwan University, TAIWAN

## Abstract

Vitamin B_1_ (thiamin) deficiency is an issue periodically affecting a wide range of taxa worldwide. In aquatic pelagic systems, thiamin is mainly produced by bacteria and phytoplankton and is transferred to fish and birds via zooplankton, but there is no general consensus on when or why this transfer is disrupted. We focus on the occurrence in salmon (*Salmo salar*) of a thiamin deficiency syndrome (M74), the incidence of which is highly correlated among populations derived from different spawning rivers. Here, we show that M74 in salmon is associated with certain large-scale abiotic changes in the main common feeding area of salmon in the southern Baltic Sea. Years with high M74 incidence were characterized by stagnant periods with relatively low salinity and phosphate and silicate concentrations but high total nitrogen. Consequently, there were major changes in phytoplankton and zooplankton, with, e.g., increased abundances of Cryptophyceae, Dinophyceae, Diatomophyceae and Euglenophyceae and *Acartia* spp. during high M74 incidence years. The prey fish communities also had increased stocks of both herring and sprat in these years. Overall, this suggests important changes in the entire food web structure and nutritional pathways in the common feeding period during high M74 incidence years. Previous research has emphasized the importance of the abundance of planktivorous fish for the occurrence of M74. By using this 27-year time series, we expand this analysis to the entire ecosystem and discuss potential mechanisms inducing thiamin deficiency in salmon.

## Introduction

Large numbers of wild animals from a wide range of taxonomic groups in several ecosystems show signs of vitamin B_1_ (thiamin or thiamine) deficiency [1–7]. In addition to mortality, thiamin deficiency can also result in compromised health, altered behavior and reproductive failure, thus potentially leading to population decline [4, 8–10]. Thiamin is a crucial micronutrient for all organisms and has various central cellular functions (for a review, see Kraft and Angert [11]). Thiamin deficiency was recently identified as a threat to global biodiversity [8], but it is not well known why and under which conditions thiamin deficiency develops in different taxonomic groups. Hence, here we focus on the abiotic and biotic conditions that prevail during periods of high and low occurrence of thiamin deficiency in salmon.

Thiamin deficiency has been especially well quantified in salmon. In the Baltic Sea, this species displays year-to-year fluctuations in the incidence of a thiamin deficiency syndrome called M74, which is reported as the proportion of females producing offspring with M74. M74 leads to offspring mortality during the yolk-sac fry stage [1, 6, 7, 9, 12], and as many as 70–80% of the females produce offspring with the syndrome in some years, leading to nearly 100% mortality in the offspring [13]. Salmon with thiamin deficiency typically exhibit discoloration of the skin and internal organs and neurological symptoms [1, 14]. It has been possible to remediate affected fish by administering thiamin [15], which indicates a link between the availability of the vitamin in the ecosystem and the deficiency syndromes. Previous research has not been able to connect the deficiency syndromes in salmon and sea birds to contaminants, genetic factors or infectious agents [14, 16–18].

**Fig 1:**
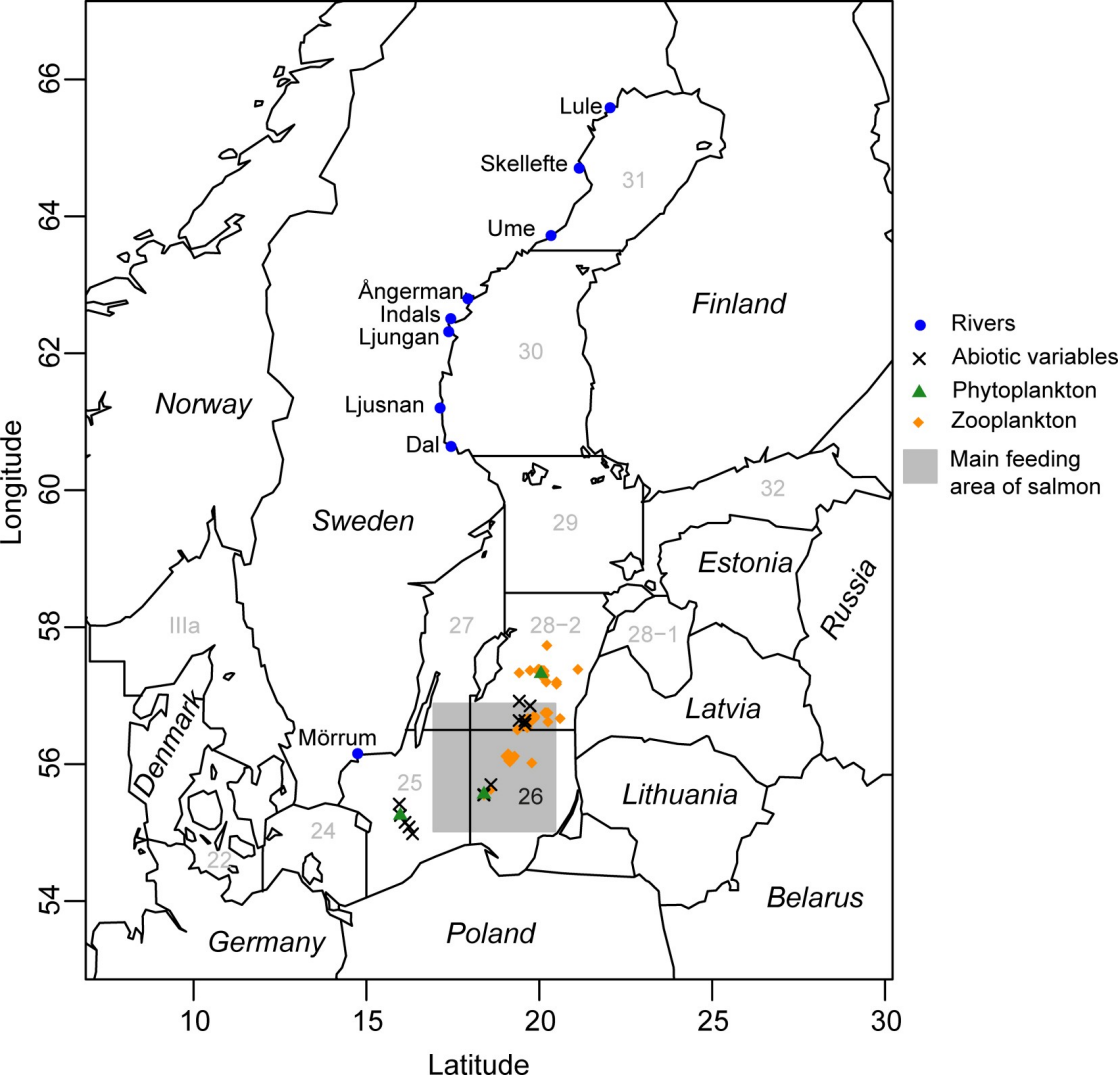
Map indicating Swedish rivers (blue) with data on M74 incidence, sampling locations for abiotic (X) and biotic variables (green, orange), ICES areas and the main feeding area (gray) for salmon.

Salmon populations in the Baltic Sea reproduce in freshwater streams, and juveniles spend a couple of years in this habitat before migrating to the brackish Baltic Sea, where they feed and grow for one to three years before they return to their native stream (Fig 1) [19]. A large proportion of these northern spawning populations migrate to the southern part of the Baltic Sea for feeding, primarily to the southern part of the Eastern Gotland Basin [20–24]. The clupeids herring (*Clupea harengus*) and sprat (*Sprattus sprattus*) constitute the main dietary items of salmon during this period [25, 26]. A diet containing a large proportion of fatty prey fish (especially juvenile sprat) has been proposed to result in a lower supply of thiamin per unit of energy [6, 7]. However, the diet of salmon had a lower proportion of sprat during years with a high incidence of M74 in the 1990s compared to a low-incidence period in 1959–1962 [25, 26]. Recently, thiamin deficiency syndromes in salmon were also connected to feeding area and type of prey by fatty acid signature analysis [27]. In the Great Lakes, the thiamin deficiency syndrome EMS (early mortality syndrome) has been proposed to be related to the introduction of a thiaminase-rich prey species, alewife (*Alosa pseudoharengus*) [28]. Thiaminase is an enzyme that breaks down thiamin, potentially causing thiamin deficiency [29]. In the Baltic Sea, herring has approximately ten times higher thiaminase activity than sprat, at levels corresponding to those in salmon guts [30, 31]. While correlations between the abundance of sprat (especially those of small size) and M74 have been found previously [7, 26], we expand the analysis to the entire ecosystem to investigate whether sprat consumption is the only parameter correlated with the M74 outbreaks.

Sprat and herring feed on zooplankton (mainly copepods) that in turn feed on the major vitamin producers in the sea, i.e., phytoplankton and bacteria [32]. Hence, all higher trophic levels depend on the thiamin produced by primary producers and the transfer of this water-soluble micronutrient through the ecosystem. In addition, not all bacteria and phytoplankton produce thiamin but need an external source of thiamin or its precursors. This auxotrophy level is very variable among phytoplankton and bacterial genera; older estimates suggest a level of auxotrophy of approximately 30%, but newer analyses suggest that this value is underestimated [32–38]. Hence, the overall production and transfer of thiamin in the aquatic food web is likely dependent on the structure and trophic interactions of the entire food web.

The Baltic Sea is heavily influenced by anthropogenic stressors that affect ecosystem structure and function [39, 40], and regime shifts have reshaped all trophic levels of the food web, having such effects as changing the species composition of phytoplankton, zooplankton, planktivorous fish and predatory fish [41–45]. Hence, our main hypothesis is that M74 outbreaks are associated to changes in the food web structure. These changes would then affect the quality and transfer of food items in terms of fatty acids, vitamins and pigments from the producers at lower trophic levels to higher trophic levels [6, 27, 46, 47] but this latter hypothesis is not within the scope of this study. Hence, the lack of an understanding of the abiotic and biotic conditions during M74-outbreaks calls for an analysis of whether large-scale changes at multiple levels of the ecosystem affect the occurrence of deficiency syndromes among top predators.

Such a large-scale analysis, to the best of our knowledge, has not been performed previously, and here we compile an extensive dataset of abiotic and biotic parameters covering multiple trophic levels and important physical parameters in the main feeding area of Baltic salmon across time and space. The aim is by assessing the structure of the food web over time to identify the underlying environmental factors associated with the incidence of deficiency syndromes. Hence, for the first time, we test the hypothesis that deficiency syndromes are associated with large-scale changes in abiotic and biotic conditions in the ecosystem.

## Methods

### Data

**Fig 2:**
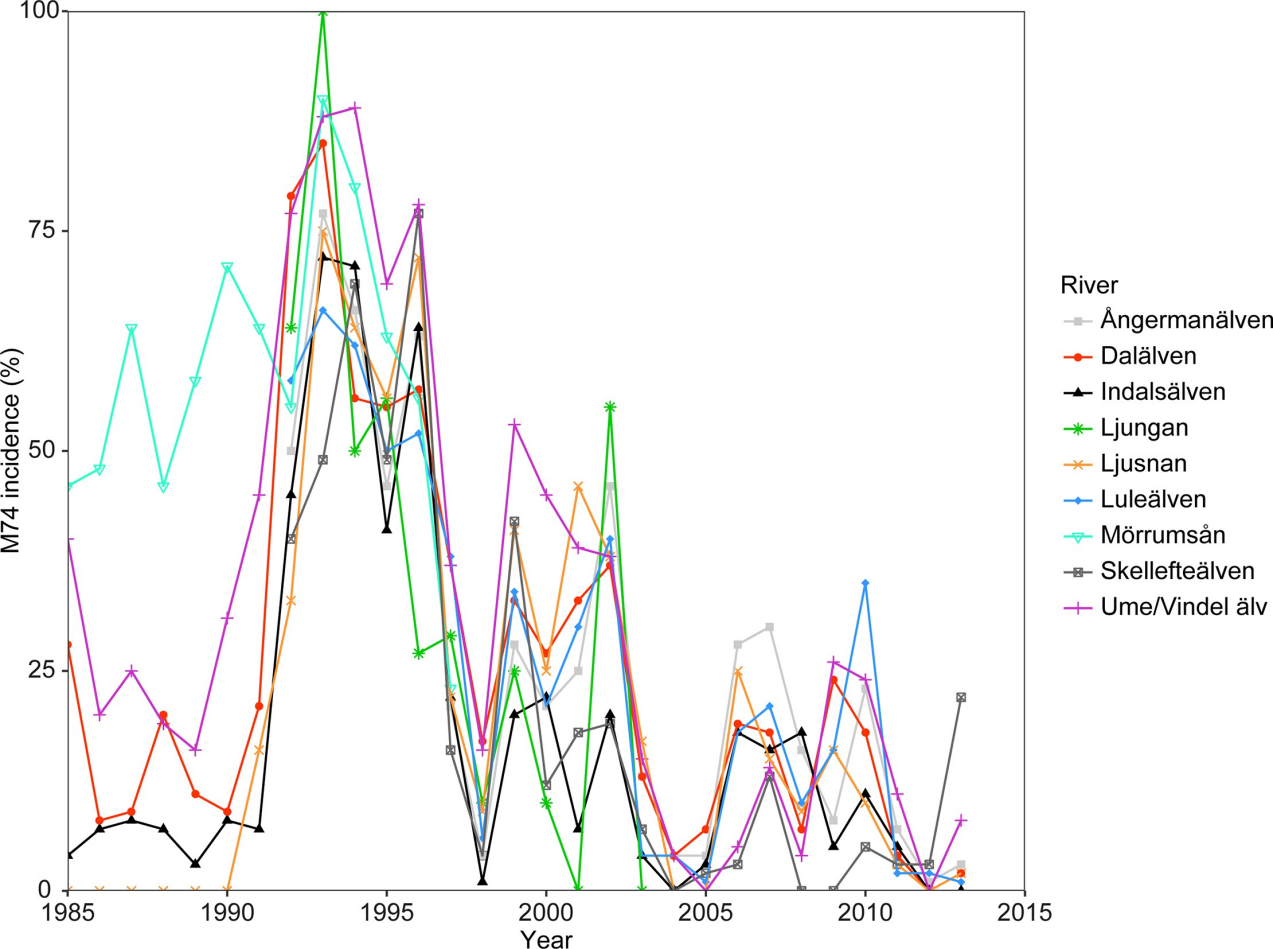
M74 incidence as the percentage of salmon females with the syndrome in Swedish spawning rivers. Data from ICES 2014 [13].

M74 was first detected in 1974, but regular monitoring of its incidence only began in the 1980s. Hence, data on M74 incidence were collected from published reports covering the period of 1985–2013 [13]. Only data from Swedish M74 monitoring programs were included to ensure that comparable sampling methods were used. All rivers except the Mörrumsån displayed high interriver correlations (Figs 1 and 2). Mörrumsån was the only sampled river entering the southern part of the Baltic Sea; although also displaying a peak in M74 incidence in the 1990s, this peak did not correlate with the M74 incidence observed in the northern populations (Spearman’s rho 0.03–0.57; p > 0.05). Hence, the average M74 incidence in all rivers except the Mörrumsån river was calculated and further used in the analysis.

Mark and recapture studies suggest that the main feeding areas of adult Baltic salmon from the Gulf of Bothnia are located primarily in the southern part of the Eastern Gotland Basin (International Council for the Exploration of the Sea (ICES) area 26) and, to a lesser extent, in the Bornholm Basin and the Eastern Gotland Basin (ICES areas 25 and 28–2, respectively). Hence, environmental data from areas 25, 26 and 28–2 (Fig 1) were selected. Biotic and abiotic variables were chosen based on the following criteria: (i) representativeness for specific ecosystem components, (ii) number of annual data points, and (iii) length of the covered period (S1 Fig). Abiotic environmental data were represented by nutrient concentrations and hydroclimatic variables measured from samples taken from the bottom to surface at stations similar to those used to measure the biotic variables (Fig 1, S1 Fig, S5 Table). The abiotic data originate from the Svenskt HavsARKiv (SHARK) database hosted by the Swedish Meteorological and Hydrological Institute (SMHI, publicly available). As there were often several nutrient measurements per station and quarter, these were averaged for the i) whole water column, ii) surface water in the upper 10 m layer and iii) bottom (S1 File). Data on key food-web components in the phytoplankton, zooplankton and fish communities were included to represent the biotic part of the pelagic ecosystem. Biomasses of different phytoplankton classes (S5 Table) were used to account for changes in the phytoplankton taxonomic composition and the total phytoplankton biomass. The phytoplankton data originate from the Baltic Marine Environment Protection Commission—Helsinki Commission (HELCOM) database hosted by ICES (publicly available). The phytoplankton data were collected and analyzed according to the HELCOM manual [48]. Zooplankton were represented by the biomass values for different zooplankton groupings (S5 Table) and were derived from a database of the Institute of Food Safety, Animal Health and Environment (BIOR) as well as from data collected within the Polish National Monitoring Programme. Samples were collected and processed according to the procedures described in [49, 50]. Two fish species are ecologically important as prey for salmon in the Baltic Sea, i.e., sprat and herring [51]. To characterize the demography of sprat and herring, the biomasses for age 1, age 2+ and all individuals in quarter 4 from the Baltic International Acoustic Survey (ICES—the Baltic International Fish Survey Working Group) were used. The biomass of each age group was estimated by multiplying its abundance by its average weight during the same survey (S5 Table). The biomass (catch per unit of effort, kg/h) of eastern Baltic cod (*Gadus morhua*) (< 30 cm and ≥ 30 cm) in quarter 1 from the Baltic International Acoustic Survey (ICES—the Baltic International Fish Survey Working Group) was used (S5 Table).

For all variables, the mean value for four quarters was calculated (1 = January-March, 2 = April-June, 3 = July-September and 4 = October-December), but due to the selection of ecologically relevant periods and missing data for some sampling periods, only data from quarters 2 and 3 for phytoplankton and zooplankton and 4 for fish (fish estimates are only available once per year, quarter 4) were used in the final analysis (quarter 1 was included when combining the data for areas 25, 26 and 28–2; S1 File, S1 Fig). Quarters were used to avoid spurious correlations created by seasonal variability and we focus on inter-annual variability within one quarter to study how M74 incidence varies over time during different seasons. Thus, the datasets used contain ecologically relevant periods, such as the spring and summer blooms of phytoplankton, the most productive and abundant periods of zooplankton; the effects of this spring and summer productivity can be observed in the following winter in fish. In addition, we assumed that during quarter 4, the fish occur in the offshore areas of the Baltic proper so that estimates are representative for herring and sprat populations. Overall, there were 29 years of M74 data, but other variables were missing data in some years, so when combining all variables, the data matrix included 21 years (1984–87, 1990–1995, 1999, 2002–2005, 2007–2008, 2010–2013) and contained time series of 101 variables (59 abiotic and 42 biotic) for area 26 (S1 Fig). Reliable estimates of the cod population for separate areas in the Baltic Sea only occur from 1991 onwards; thus, a separate analysis was conducted focusing on the period 1991–2013 and including time series of 104 variables (59 abiotic and 45 biotic) (S1 File).

### Statistical analyses

For the data analyses, all biotic biomass data were square root transformed to reduce the relationship between the mean and the variance and to weight the contributions of “common” and “rare” species in the multivariate representations. Datasets containing abiotic and a combination of biotic and abiotic variables were normalized to adjust the values measured on different scales to a common scale. For the abiotic data, if one to two values were missing from the dataset, they were replaced by variable averages using the “missing” command in Primer 7 [52]. To construct a dataset covering a larger study area, we combined all data from all three ICES areas, and the similarity among these areas was tested using a data matrix covering all 15 common years (1984–1987, 1990, 1995, 2002–2005, 2007, 2010–2013; S1 File). For all datasets (biotic, abiotic and biotic+abiotic), all analyses were conducted twice: a) first matching each year and b) second using a delay of one year to account for migration and feeding history. The significance level of all tests was set at 95% (p≤ 0.05).

Principal coordinate analysis (PCO) followed by canonical correlation analysis based on distances (CAP; [53, 54]) of the yearly average M74 incidence were conducted in PRIMER 7, with default Euclidian distance (abiotic and abiotic + biotic) and Bray-Curtis dissimilarity (biotic) as the distance measures. Initially, separate CAP analyses for the main feeding area for Baltic salmon (ICES area 26) were performed for the abiotic and biotic variables and all variables combined (abiotic + biotic) independently. The separate CAP analyses of the abiotic and biotic datasets allowed the inclusion of a larger range of variables for the analysis due to the missing sampling periods for different variables. Afterwards, all data from all three areas were combined and averaged, and a PCO followed by a CAP were run on the entire merged dataset (S1 File). In the CAP analysis, the program determined the appropriate number of dimensions (m) with the lowest misclassification error (%) to be included in the principal coordinate and discriminant analysis. However, if m was too similar to the number of samples, the lowest value of m with the highest accuracy was chosen. The highest number of permutations possible (9999) was used. Key species and physical drivers affecting the significance of the CAP outcome were determined from the output using Spearman's rank correlations. Spearman’s rank correlations were also used to identify the general relationships between two variables (e.g., M74 incidence and the key abiotic and biotic variables identified in the CAP analysis) and test these associations. Spearman’s rank correlation was chosen because it measures the extent to which one variable tends to increase or decrease as the other variable increases without requiring a linear relationship between them. The strengths of the correlations were categorized into moderate (0.4–0.6) and strong (0.7–0.9) according to Dancey and Reidy [55].

## Results

### Long-term trends in M74 incidence

M74 incidence has fluctuated over recent decades, with peak incidence in all rivers during the 1990s (Fig 2). All populations of salmon feeding in the Baltic Sea and reproducing in the rivers entering the northern part of the Baltic Sea (Gulf of Bothnia) showed a similar intensity of M74 deficiency syndrome incidence at the same time (Spearman’s rho 0.61–0.95; p < 0.05).

### Years with high versus low M74 incidence

**Fig 3:**
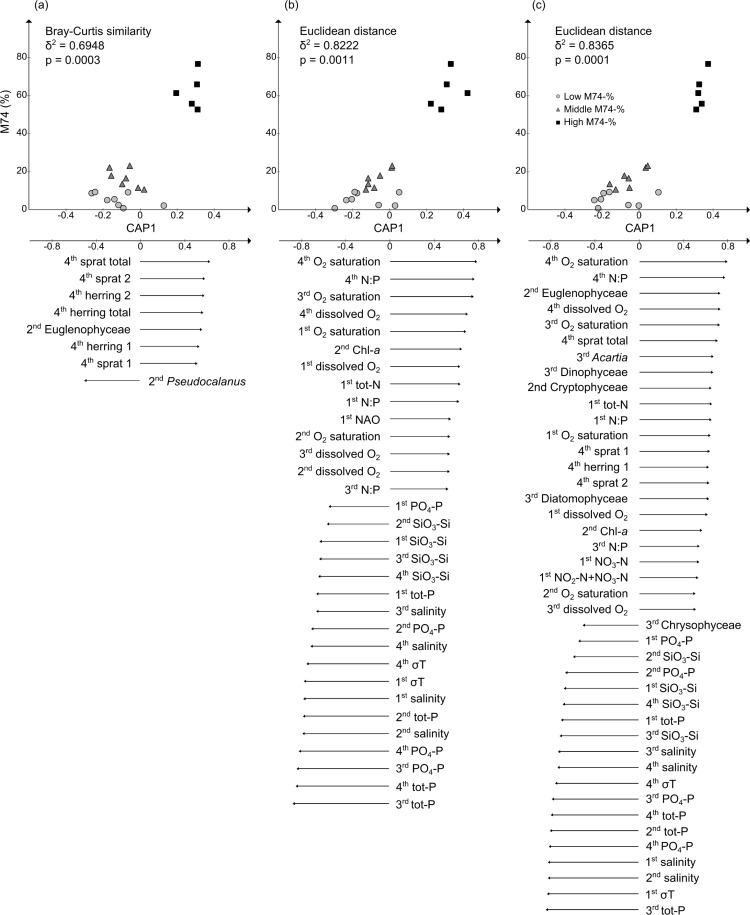
M74 incidence as a function of CAP1 (PCO followed by CAP) for (a) biotic, (b) abiotic and (c) combined biotic and abiotic variables (upper panel). Years are shifted one year to account for feeding and migration. Variables with a moderate or strong correlation (>0.4) with the CAP1 axis are indicated with arrows (lower panel, length of the arrow relates to correlation strength). 1^st^, 2^nd^, 3^rd^ and 4^th^ refer to quarters (January-March, April-June, July September and October-December, respectively).

M74 incidence and environmental variables (biotic and abiotic) in the main feeding area of salmon in the Baltic Sea, i.e., the southern part of the southeastern Gotland Basin (Fig 1; ICES area 26), fluctuated over the study period, and the yearly mean M74 incidence was significantly correlated with the variables summarized by the first canonical principal coordinate, i.e. CAP1 (Fig 3, S7 Fig). Significant canonical correlations were found for the biotic, abiotic and combined biotic and abiotic variables when analyzed against the yearly mean M74 incidence. The correlations were generally stronger when the data were shifted one year, which supports the assumption that salmon reproductive success is affected by the abiotic and biotic conditions in the year preceding migration from the southern Baltic Sea to northern spawning rivers, in comparison to the unlagged time. M74 incidence was correlated to the different variables divided into different seasons. This was done in order to avoid spurious correlations driven by seasonal variability and this enables us to study how incidence correlates to different variables over time within seasons. This facilitates the interpretation since some variables are more important during spring, summer, fall and winter, respectively.

According to the discriminant analysis for area 26, years with high M74 incidence (>30%) showed a significant grouping that was distinctly separate from the other years (intermediate incidence: 10–30%, low incidence: <10%, S1 Table); again, this pattern was similar for both the unlagged and 1-year lagged time series. However, the confidence with which the years with high M74 incidence were grouped together was 100% when a one-year delay was included, and the misclassification error was notably higher when matching years were used (>35%) and when other areas (Fig 1) close to the main feeding area were included in the analysis (S2 Table). Thus, for the remaining results section, we only present data from area 26, and analyses for other parts of the southern Baltic Sea are available in the S1 File. Furthermore, the values shown are averages from the entire water column unless mentioned otherwise.

### Abiotic parameters

The abiotic factors during years with a high incidence of M74 were positively correlated with total nitrogen (TotN), nitrate, and nitrite during quarter 1 (Fig 3, S7 and S8 Figs). For example, when the lagged time series were used, average inorganic nitrogen in quarter 1 averaged 3.8 ± 0.6 μmol/l during the years with low incidence and 5.2 ± 0.6 μmol/l during the years with high incidence. In contrast, M74 incidence showed moderate to strong negative correlations with the phosphate and total phosphorus (TotP) concentration throughout the year. During the summer months of high incidence years, the phosphate and TotP levels were low (0.2–0.4 μmol/l and 0.6–0.7 μmol/l, respectively), whereas the levels were significantly higher, varying between 0.5–0.8 μmol/l and 0.8–1.2 μmol/l, respectively, during years with low M74 incidence. A positive correlation between the M74 incidence and TotN:TotP (molar basis) ratio was also detected, especially from October to June. For example, during quarters 1 and 4, the TotN:TotP ratio was 5.8 ± 1.1 on average during the high-incidence years of 1991–1995 and 3.1 ± 1.1 during the years when the M74 incidence was lower than 10%. A moderate to strong positive correlation was also detected between M74 incidence and oxygen saturation and concentration throughout the entire year. For example, oxygen saturation fluctuated between 90.0 and 106.9% during years with high M74 incidence and between 68.9 and 94.4% during the years with low M74 incidence (S8 Fig). Furthermore, a negative correlation was detected between M74 incidence and silica and salinity. The highest silica concentration, up to 21.5 μmol/l, was measured during winter in the years with low M74 incidence, which contrasts with the highest concentration of 16.9 μmol/l being measured during years with high M74 incidence. In general, during the winter-spring-summer transition, the silica concentration varied between 11.9 and 21.5 μmol/l during years with low M74 incidence and between 9.4 and 16.9 μmol/l during years with high incidence. Years with low M74 incidence also showed higher depth-integrated mean salinity throughout the years compared to years with high M74 incidence, with values of 8.0 ± 0.1 and 7.5 ± 0.2, respectively, and the largest difference was identified in late spring (9.0 ± 0.2 and 7.6 ± 0.2, respectively).

### Biotic parameters

Regarding phytoplankton, only the springtime biomass of class Euglenophyceae (2.0 ± 2.7 μg/dm^3^ and 21.6 ± 15.8 μg/dm^3^ for low and high M74, respectively) showed a positive correlation with M74 incidence when the biotic variables were analyzed alone. In contrast, when both biotic and abiotic variables were combined for the analysis, the springtime biomass of the class Cryptophyceae (39.8 ± 21.5 μg/dm^3^ and 70.3 ± 31.5 μg/dm^3^, respectively) and the summer biomass of Dinophyceae (47.5 ± 31.2 μg/dm^3^ and 252.2 ± 155.4 μg/dm^3^, respectively) and Diatomophyceae (19.4 ± 18.6 μg/dm^3^ and 54.6 ± 56.8 μg/dm^3^, respectively) showed a strong or moderate positive correlation with M74 incidence (S8 Fig). Furthermore, there was a negative correlation between M74 incidence and Chrysophyceae biomass (12.3 ± 21.6 μg/dm^3^ and 0.06 ± 0.1 μg/dm^3^, respectively) during summer.

Regarding zooplankton, the springtime *Pseudocalanus* spp. biomass showed a negative correlation with M74 incidence while the summer stock of *Acartia* spp. (S8 Fig) showed a positive correlation when the lagged time series were analyzed. Despite the observation of no clear trend in the *Acartia* spp. to *Pseudocalanus* spp. ratio, the *Pseudocalanus* spp. stock in spring was significantly higher during years with low M74 incidence than in years with high incidence (5609 ± 2687.9 μg/m^3^ and 3445 ± 2497.1 μg/m^3^, respectively), while *Acartia* spp. showed the opposite pattern (3575 ± 4471.9 μg/m^3^ and 8248.7 ± 5098.5 μg/m^3^, respectively).

Within the biotic time series, the strongest positive correlation with M74 incidence was found for sprat biomass, including both the biomass of juveniles and older sprat as well as total biomass (S8 Fig). The standing stock of total sprat biomass was 40% lower during the years of low M74 incidence compared to years with high M74 incidence (235,075 ± 156,035 and 585,244 ± 70,101 tons, respectively). Additionally, the biomass of the total herring stock and the abundance of juvenile herring showed a positive correlation with M74 incidence; similarly, the total herring biomass was 39% lower during the years of low M74 incidence (201,958 ± 48,742 and 518,859 ± 174,097 tons, respectively) (S8 Fig). Data on cod are only available for a few years of the time series and were generally not included in the analyses. When only years with cod data (1991–2013) were included in the analysis, the (positive) correlations were similar for more environmental parameters than in the full analysis in the cases of both matching years (t^2^ = 0.80, p = 0.002) and when one year of migration and feeding history was taken into consideration (t^2^ = 0.92, p = 0.0006; S1 File). However, in both cases, only the biomass of small cod (<30 cm) showed a positive correlation with M74 incidence (canonical correlations of 0.62 and 0.6, respectively).

## Discussion

M74 is periodically causing high mortality among Baltic salmon offspring and it has been shown to be caused by a deficiency in thiamin [1, 56] but the question remains as to why this deficiency arises. One hypothesis is that changes in abiotic conditions constrain the transfer of thiamin in the food web from the producers (i.e. phytoplankton and bacteria) to the consumers [47]. The second hypothesis is that M74 arise when salmon eat proportionally more small-sized sprat compared to large sprat and herring [7, 26]. The small sized sprat have relatively low concentrations of thiamin per energy content compared to other clupeids [6, 9, 27]. Yet another hypothesis is that the prey fish of salmon (sprat and herring) contain a thiamin degrading enzyme called thiaminase reducing the uptake of thiamin [30, 31]. These three hypotheses are discussed below in relation to the present data set.

**Fig 4:**
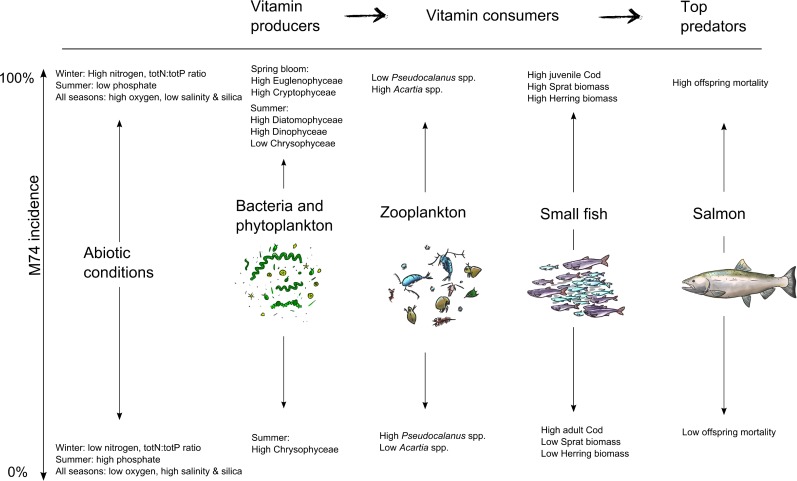
Schematic illustration of how salmon thiamin deficiency syndrome is strongly influenced by large-scale changes in the abiotic and biotic variables in the ecosystem.

Our findings demonstrate that salmon thiamin deficiency syndrome (M74) is strongly associated with large-scale changes in certain abiotic and biotic variables in the Baltic Sea ecosystem (Fig 4). Based on our long-term data, we demonstrate that the peak incidence of deficiency syndromes occurs when the main feeding area of salmon undergoes periods of freshening (lower salinity) with relatively high availability of nitrogen and concurrent changes in the phytoplankton, zooplankton and fish communities. Overall, this result supports the first hypothesis described above suggesting that thiamin deficiency is caused by changes in abiotic factors with concurrent changes in the food web structure leading to a reduction in the production and transfer of thiamin in the food web. The outcome of changes in abiotic conditions, such as nutrient availability, has recently been tested in a modelling study and it was shown that bottom-up effects can constrain the transfer of thiamin from producers to consumers in aquatic food webs [47]. Hence, the changes in abiotic conditions and consequent changes in the species composition and size distribution of primary producers, that have occurred in the Baltic Sea, could have constrained the flow of thiamin from lower to higher trophic levels of the system. However, not only bottom-up factors such as nutrients seem to play a role since it has also been shown that M74 incidence is correlated with the stock size of sprat [7, 26], with the suggested cause of M74 being salmon consumption of small individuals with a relatively low thiamin concentration per unit of fat content [6, 27]. Our data supports that M74 is most severe during periods with high abundance of planktivorous fish but correlations occur, not only for sprat, but also for herring. Hence, a constrained flow of thiamin from the primary producers such as phytoplankton and bacteria up to fish together with a high abundance of planktivorous fish seem to increase the risk of M74 in salmon. Recent modelling studies also confirm these two mechanisms to explain a reduced concentration of thiamin in clupeids [47]. However, other studies question the importance of specifically young sprat (compared to other diets of clupeids) for the development of M74 since the diet of salmon in fact contained a lower proportion sprat during years of high M74 incidence in the 1990s in comparison to a low-incidence period in 1959–1962 [25]. Furthermore, the thiamin levels in both sprat and herring are mostly above the dietary requirements of salmon [6, 57], suggesting that we lack a fundamental understanding of additional important mechanisms inducing thiamin deficiency in the ecosystem in general and in salmon in particular. The third hypothesis for development of M74 and thiamin deficiency is the occurrence of a thiamin degrading enzyme called thiaminase in the diet of salmon. Here, we show that outbreaks of M74 were associated with large-scale changes in abiotic and biotic factors changing the entire food web but we cannot determine if these conditions results in a higher risk for a salmon diet which is rich in thiaminase. Future studies need to quantify the relative contribution of the thiaminase-hypothesis in relation to a constrained transfer of thiamin in the food web and the effects of consumption of thiamin poor clupeids for the overall thiamin and M74 status of salmon.

### Salmon migration and M74

We show high interriver correlations in terms of M74 incidence among northern rivers, suggesting that the salmon populations spawning in different rivers are exposed to common factors that episodically result in elevated M74 incidences. Previously, it has been shown that the southern part of the Eastern Gotland Basin (subdivision 26) is in the main feeding area of adult salmon during their feeding phase and that the majority of the fry that hatch in the northern rivers migrate to this part of the Baltic Sea after a two-to-three-year freshwater phase, e.g., McKinnel and Lundqvist [21]. After a few years in the Baltic Sea, salmon migrate back to their spawning rivers while reducing feeding and mainly relying on stored energy during the migration [19]. Their feeding history is therefore important in determining their reproductive success and could explain why the relationship between the deficiency syndrome was stronger in the main feeding area (subdivision 26) than in the surrounding areas (subdivisions 25 and 28–2). Furthermore, the environmental variables were more strongly correlated with M74 incidence when the variables were shifted by one year, i.e., taking one year into account to compensate for feeding history and migration.

### Abiotic factors

Interestingly, the intensity of the incidence of the deficiency syndrome follows the frequency of the so-called major Baltic inflows (MBIs), which are events that occur when saline water flows into the Baltic Sea from the Atlantic, followed by periods in-between inflows with stagnation and relatively less saline waters (Fig 2; MBIs occurred in 1976, 1993/1994, 2002/2003, 2010 (weak) and 2014; [58]). Typically, periods with high M74 incidence were characterized by relatively low salinity and high oxygen levels. These periods were mainly caused by the occurrence of a deeper and weaker halocline, where fresh and oxygen-rich water extend deeper down, leading to an overall lower salinity and higher oxygen level when averaging over the entire water column. These types of physical conditions are typical of stagnant periods in the Baltic Sea. The changes in the hydrographic conditions can be recognized most distinctly on the basis of variations in nutrient concentrations [58], which is in line with our results. For example, phosphate and silicate concentrations were lower in periods with high M74 incidence, whereas TotN concentrations (as well as those of nitrate and nitrite) were higher. Differences in the hydrographic and chemical parameters [59–62] consequently relate to the living conditions for the biota and the overall trophic structure of the ecosystem [63]. Changes in abiotic factors such as nutrient availability and light conditions have been shown in modelling studies to constrain the transfer for thiamin from lower to higher trophic levels [47] suggesting that the abiotic changes observed during high incidence years could have affected the thiamin status and M74 occurrence in salmon.

### Phytoplankton

The incidence of M74 in salmon and phytoplankton biomass during the productive period in the Baltic Sea showed many significant relationships, demonstrating the importance of phytoplankton community composition for the rest of the Baltic Sea ecosystem. Several recent studies have reported shifts in phytoplankton biomass as well as in overall species composition in relation to changing environmental conditions [45, 63, 64], and these results are in line with those from our study. We observed high biomass of Euglenophyceae and Dinophyceae during high-incidence years. A low Diatomophyceae to Dinophyceae ratio has been suggested to indicate a generally deteriorated environmental status [62], and shifting dominance in the phytoplankton community could also indicate changes in food quality in terms of fatty acids [60] and an overall reduction in food quality in the Baltic Sea during years of high M74 incidence. However, the importance of Diatomophyceae, Dinophyceae, and other phytoplankton taxa as food items for zooplankton has not been clearly established. For example, Diatomophyceae contain relatively high concentrations of fatty acids, which induce high egg production in copepods, but at the same time have other substances that could lead to reduced hatching and survival of eggs and juveniles [59, 60].

The concentration of thiamin in the water of the Baltic Sea is not known, but levels of dissolved thiamin in natural systems are generally low (pM), indicating a larger demand than supply in the world’s oceans [34, 61, 65–67]. Several marine bacteria and phytoplankton are thiamin (or its precursors) auxotrophs that do not synthesize their own thiamin [32–36, 38, 68, 69]. Furthermore, the level of auxotrophy is quite variable among phytoplankton phyla, ranging from 0% to 83% in the investigated phytoplankton species [32, 33], but it is not known how auxotrophy affects the transfer of thiamin in the food web. Apart from relatively high abundance of Euglenophyceae and Dinophyceae during high M74 incidence years there were also relatively more Cryptophyceae, Diatomophyceae and fewer Chrysophyceae during some seasons. Of the few phytoplankton species that have been screened for thiamin content, cyanobacteria have the highest thiamin levels, while Dinophyceae and Diatomophyceae have intermediate levels, and phytoplankton also show a high plasticity in thiamin content in response to abiotic changes [70, 71]. Recently, the seasonal dynamics of the thiamin content of seston (phytoplankton, bacteria and detritus) in the Baltic Sea were investigated. Thiamin levels were higher in relatively small seston and showed elevated levels during the summer months, when Prymnesiophyceae and Cyanophyceae composed a large proportion of the phytoplankton community [72], but these two phytoplankton classes do not emerge as significant in the present study. Hence, changes in species composition and differential thiamin production within species in response to abiotic conditions can lead to reduced availability of this micronutrient for higher trophic levels. But the knowledge on thiamin concentrations in different phytoplankton taxa is too limited to determine if the changes in species composition observed here had an effect on overall thiamin transfer. However, recent modelling suggests that shifts in the size structure of the phytoplankton community can affect the overall transfer of thiamin in the ecosystem [47]. For example, thiamin transfer has been suggested to be constrained in food webs that are dominated by pico-sized phytoplankton compared to micro-sized phytoplankton [47]. This could explain the strong relationships between the M74 deficiency syndrome in salmon and the abundance of some classes of phytoplankton, but the transfer of thiamin from producers to top predators must be better understood and modeled to further elucidate these correlations.

### Zooplankton

The transfer of thiamin from phytoplankton through zooplankton to higher trophic levels is not fully understood. A weak, albeit significant, correlation between seston thiamin and zooplankton thiamin concentrations has been found in field studies [72]. That thiamin concentrations in zooplankton are only weakly proportional to the concentrations in their food items likely occurs because zooplankton selectively feed on preferred prey items [73, 74]. For example, filamentous cyanobacteria are relatively rich in thiamin, but this thiamin is not readily transferred to zooplankton because filaments are difficult to ingest [71]. Our results suggest that high M74 incidence is associated with high biomass of *Acartia* spp. and low biomass of *Pseudocalanus* spp. and that these zooplankton are in turn consumed by the main planktivorous fish, sprat and herring. *Pseudocalanus* spp. abundance has generally decreased in the southern Baltic Sea since the late 1980s [41]; these species are generally larger than *Acartia* spp. and are therefore selectively preyed upon by small sprat and herring [75]. *Acartia* spp. were recently reported to have a higher thiamin content than both *Temora* sp. and *Pseudocalanus* sp. [72]. Altogether, a high abundance of *Acartia*, which could be the result of avoidance by both herring and sprat [75], seems to lead to inefficient transfer of micronutrients from zooplankton to planktivorous fish, but the actual transfer efficiency and bioavailability of thiamin among trophic levels is not known and needs further study.

### Fish and trophic dilution of thiamin

Years with higher abundances of the planktivorous herring and sprat were also followed by years of higher M74 incidence. Such a relationship has been suggested previously [6, 7, 9], and thiamin deficiency has been proposed to arise due to high consumption by salmon of small sprat containing insufficient amounts of thiamin in relation to energy content [6, 9]. Recent modeling also shows that transfer of thiamin in the food web is constrained during periods of high clupeid fish abundance [47]. However, the thiamin concentrations in both herring and sprat are generally higher than the assumed demand of salmon, suggesting that we do not fully understand the mechanism involved in the development of M74 [6, 57]. The relationship among herring, sprat and M74 incidence has also been suggested to be related to the high thiaminase levels present in herring in particular [30, 31], which would further reduce the thiamin levels in salmon. Thiaminase activity has also been shown to exhibit seasonal, annual and spatial variation among prey and predator fishes in the Great Lakes [76, 77] and can also increase over short time periods [30]. In the present study, the results show positive relationships between salmon deficiency syndromes and both herring and sprat of all size classes as well as small-sized cod, indicating that both prey species and other piscivorous fish (i.e., cod) are important in determining the incidence of M74 in salmon, but the mechanisms underlying these relationships are not known. Furthermore, we detected large-scale changes in hydrography and in multiple trophic levels during high-incidence years, indicating that an increase in small-sized sprat alone may be a simplified explanation for the outbreak of M74 deficiency syndrome. In biota there is a trophic dilution of thiamin with median concentrations of 932 nmol gC^-1^ in seston (i.e. bacteria, phytoplankton etc.), 334 nmol gC^-1^ in mesozooplankton [72] and concentrations ranging from approximately 20–100 nmol gC^-1^ in clupeids (assuming 12.5% carbon mass of fresh body mass; [6, 78]). Hence, the main prey items for salmon, i.e. clupeids, have in the order of 3–16 times lower thiamin concentrations compared to zooplankton. Modelling has shown that changes in the size distribution of phytoplankton leads to large changes in the overall transfer of thiamin in the food web [47]. For example, if microphytoplankton dominate the thiamin production, then ~2% of the thiamin reach clupeids, whereas, if small sized picophytoplankton dominate the system only ~0.0005% of the thiamin reach clupeids [47]. When salmon are accessing thiamin from the diet (i.e. mainly clupeids) it has also been argued that the risk of thiamin deficiency increases when consumption of juvenile sprat is high since these contain a lower supply of thiamin per unit of energy [6, 9, 27]. Finally, during the transfer of thiamin from clupeids to salmon there is also a risk of thiamin breakdown if the enzyme thiaminase is present [30, 31] but there are no studies that evaluate how different enzyme activities change the overall transfer of thiamin to salmon given the thiamin concentration available in the prey.

In conclusion, we demonstrate that the incidence of thiamin deficiency syndromes is related to large-scale changes in certain abiotic conditions and to changes in the structure of the food web in terms of both the quantity and quality of all trophic levels. The relationships between M74 incidence and environmental variables were stronger when the environmental variables were shifted one year, suggesting that the conditions prior to salmon migration for spawning are important for the occurrence of M74. Peak M74 incidences occurred when the Baltic Sea was undergoing freshening (lower salinity) with relatively low phosphate and silicate concentrations and high availability of nitrogen, and these bottom-up forces affected the complex connectivity among the trophic levels. High incidence was also present when there was a high abundance of planktivorous fish. Studies that quantify the strength of thiamin transfer from lower trophic levels via clupeids to salmon and mechanistic studies that quantify the effect of a fat-rich diet together with the potential influence of thiaminase on thiamin transfer in the ecosystem are still needed to elucidate if these different mechanisms work together to induce M74 or if one of them is more important for the overall outcome.

Finally, as it was recently suggested that thiamin deficiency is considerably more widespread and severe than previously expected, potentially causing declines in several wildlife populations, including several bird species [3, 4, 10], this study provides evidence using a long-term dataset (~30 years) that deficiency syndromes are indeed associated with large-scale whole-ecosystem changes in certain abiotic and biotic factors. This lays a strong statistical foundation for works modelling thiamin transfer in ecosystems [47] and addressing the mechanisms responsible for outbreaks of thiamin deficiency in salmon and in other species.

## Supporting information

S1 File Supplementary methods and results. (DOCX)

S1 Fig Variables included in the main analyses. Gray areas indicate missing data, and black areas indicate the presence of data.
(TIF)

S2 Fig Discriminant analysis in which biotic and abiotic variables were significantly clustered into three different groups based on the subdivision (ICES subdivision 25, 26 and 28–2). Choice of m with first squared canonical correlation: 7 with 0.9. Variables with a correlation >0.7 with the canonical axis are shown.
(PPTX)

S3 Fig Principal coordinate analysis (PCO) followed by canonical correlation analysis based on distances, (a) biotic, (b) abiotic and (c) both biotic and abiotic variables combined when the sampling years were matched together (upper panel) in ICES subdivisions 25, 26 and 28–2. The intensity of the M74 incidence (high >30%, intermediate 10–30%, and low <10%) is indicated by the shape of the data points. Choice of m with first squared canonical correlation: (a) 5 with 0.4, (b) 6 with 0.7 and (c) 6 with 0.7. Variables with moderate or strong correlations (>0.4) with the canonical axis are listed for all datasets (lower panel, arrows).
(PNG)

S4 Fig Principal coordinate analysis (PCO) followed by canonical correlation analysis based on distances, (a) biotic, (b) abiotic and (c) both biotic and abiotic variables combined when the migration time of the salmon was taken into consideration (upper panel) in ICES subdivisions 25, 26 and 28–2. The intensity of the M74 incidence (high >30%, intermediate 10–30%, and low <10%) is indicated by the shape of the data points. Choice of m with first squared canonical correlation: (a) 5 with 0.7, (b) 9 with 0.7 and (c) 6 with 0.7. Variables with moderate or strong correlation (>0.4) with the canonical axis are listed for all datasets (lower panel, arrows).
(PNG)

S5 Fig Principal coordinate analysis (PCO) followed by canonical correlation analysis based on distances, (a) same year and (b) 1-year delay (upper panel) including cod in subdivision 26. The intensity of the M74 incidence (high >30%, intermediate 10–30%, and low <10%) is indicated by the shape of the data points. Choice of m with first squared canonical correlation: (a) 5 with 0.8 and (b) 4 with 0.8. Variables with moderate or strong correlations (>0.6) with the canonical axis are listed for all datasets (lower panel, arrows).
(PNG)

S6 Fig Principal coordinate analysis (PCO) followed by canonical correlation analysis based on distances, (a) upper 10 m layer and (b) bottom layer with 1-year delay (upper panel) in subdivision 26. The intensity of the M74 incidence (high >30%, intermediate 10–30%, and low <10%) is indicated by the shape of the data points. Choice of m with first squared canonical correlation: (a) 8 with 0.7 and (b) 14 with 0.9. Variables with moderate or strong correlations (>0.4) with the canonical axis are listed for all datasets (lower panel, arrows).
(PNG)

S7 Fig Principal coordinate analysis (PCO) followed by canonical correlation analysis based on distances, (a) biotic, (b) abiotic and (c) both biotic and abiotic variables combined when matching years were used (upper panel) in subdivision 26. The intensity of the M74 incidence (high >30%, intermediate 10–30%, and low <10%) is indicated by the shape of the data points. Choice of m with first squared canonical correlation: (a) 2 with 0.5, (b) 5 with 0.7 and (c) 13 with 0.9. Variables with moderate or strong correlation (>0.4) with the canonical axis are listed for all datasets (lower panel, arrows).
(PNG)

S8 Fig Examples of significant correlations (Spearman rank) between M74 incidence and environmental variables, including salinity (a), σ_T_ (b), oxygen saturation (c), total nitrogen (d), total phosphate (e), silicate (f), Craspedophyceae (g), Euglenophyceae (h), Dinophyceae (i), *Acartia* spp. (j), herring (k) and sprat (l). Spearman´s rank correlation coefficients and number of years in top right corner of each subgraph. Significance level indicated by asterisks, p<0.0001 (****), p<0.001 (***), p<0.01 (**), p<0.05 (*). For all remaining significant correlations, see S4 Table.
(TIF)

S1 Table Results of discriminant analysis with the three different *a priori* groups: high (>30%), intermediate (30%>M74>10%) and low (<10%) M74 incidence with biotic, abiotic and combined biotic and abiotic variable datasets for ICES subdivision 26. The upper light gray panel shows the results for the matching years, and the lower dark gray panel provides the results for when the 1-year delay (salmon migration and feeding) was taken into consideration.
(DOCX)

S2 Table Results of discriminant analysis with the three different *a priori* groups: high (>30%), intermediate (30%>M74>10%) and low (<10%) M74 incidence with biotic, abiotic and combined biotic and abiotic variable datasets for ICES subdivisions 25, 26 and 28–2 combined. The upper light gray panel shows the results for the matching years, and the lower dark gray panel provides the results when the 1-year delay (salmon migration and feeding) was taken into consideration.
(DOCX)

S3 Table Results of discriminant analysis with the three different *a priori* groups: high (>30%), intermediate (30%>M74>10%) and low (<10%) M74 incidence with the surface and bottom datasets for ICES subdivision 26. The upper light gray panel shows the results for the matching years, and the lower dark gray panel provides the results for when the 1-year delay (salmon migration and feeding) was taken into consideration.
(DOCX)

S4 Table Examples of significant correlations (Spearman’s rank) between M74 incidence and environmental variables are shown in S8 Fig. The remaining significant correlations between M74 incidence and environmental variables are listed in this table. Significance level indicated by asterisks, p<0.0001 (****), p<0.001 (***), p<0.01 (**), p<0.05 (*).
(DOCX)

S5 Table Summary of variables. (DOCX)
