# Supplementary material for: Deficiency syndromes in top predators associated with large-scale changes in the Baltic Sea ecosystem
Source: PLoS One. 2020 Jan 9;15(1):e0227714. doi: 10.1371/journal.pone.0227714 (PMC6952091; doi:10.1371/journal.pone.0227714)
Supplement: S1 File — (DOCX) [file pone.0227714.s001.docx]

**Title:** Deficiency syndromes in top predators associated with large-scale changes in the Baltic Sea ecosystem

**Short title:** Deficiency syndromes in Baltic Sea salmon

Sanna Majaneva*^1,2^, Emil Fridolfsson*^1^, Michele Casini^3^, Catherine Legrand^1^, Elin Lindehoff^1^, Piotr Margonski^4^, Markus Majaneva^5,1^, Jonas Nilsson^1^, Gunta Rubene^6^, Norbert Wasmund^7^ and Samuel Hylander^1^

*Authors with equal contributions

**Corresponding author** sanna.majaneva@gmail.com, +4791154485

**Supplemental results**

**M74 incidence and environmental parameters in ICES subdivisions (25, 26 and 28-2)**

The datasets from the different areas of the Baltic Sea had slightly different coverage, with subdivisions 25 and 28-2 missing data for some variables and/or years that are available from subdivision 26 and vice versa (Fig. S1). However, an analysis comparing the variables available for all areas identified some biotic and abiotic variables that vary similarly among areas (RELATE, 25 versus 26, Rho=0.6, p=0.02; 25 versus 28, Rho=0.6, p=0.01; 26 versus 28, Rho=0.6, p=0.04). Principal coordinate analysis (PCO) followed by generalized discriminant analysis based on distances (CAP) with Euclidian distance (abiotic and abiotic + biotic) and Bray-Curtis dissimilarity (biotic) as distance measures were conducted for the three different areas as *a priori* groups based on the intensity of the M74 incidence (high >30%, intermediate 10-30%, and low <10%) and the biotic, abiotic and biotic and abiotic variables combined, grouped separately based on the area (biotic: δ^2^_1_ = 0.65, t_2_ =0.91, p < 0.001, 9999 permutations, misclassification error 31%; abiotic: δ^2^_1_ = 0.88, t_2_ =1.55, p < 0.001, 9999 permutations, misclassification error 2%; biotic+abiotic: δ^2^_1_ =0.90, t_2_ = 1.73, p = 0.001, 9999 permutations, misclassification error 0%) (Fig. S2). As expected, subdivision 25 had the highest salinity throughout the year (e.g., winter salinity in subdivision 25: 10.0 ± 0.5, subdivision 26: 8.1 ± 0.3 and subdivision 28-2: 9.0 ± 0.5) and the highest wintertime temperature (4.5 ± 0.1°C for subdivision 25, 3.6 ± 0.7°C for subdivision 26 and 4.1 ± 0.4°C for subdivision 28-2). Subdivision 26, on the other hand, had more oxygen available throughout the year than subdivision 28-2 (70 ± 4 µmol/l for subdivision 25, 80.5 ± 3.3 µmol/l for subdivision 26 and 59.6 ± 7.9 µmol/l for subdivision 28-2). Subdivisions 28-2 and 26 together were more productive in terms of total phytoplankton (4.3 ± 0.7 µg/dm^3^ for subdivision 25, 5.0 ± 0.9 µg/dm^3^ for subdivision 26 and 5.1 ± 0.7 µg/dm^3^ for subdivision 28-2), Dinophyceae in spring (3.6 ± 0.7 µg/dm^3^ for subdivision 25, 4.3 ± 0.8 µg/dm^3^ for subdivision 26 and 4.3 ± 0.7 µg/dm^3^ for subdivision 28-2) and Cyanobacteria in summer (2.5 ± 0.3 µg/dm^3^ for subdivision 25, 4.0 ± 1.2 µg/dm^3^ for subdivision 26 and 4.4 ± 0.9 µg/dm^3^ for subdivision 28-2) than the more southern part (subdivision 25). No significant variation in zooplankton or fish stocks was detected among the different subdivisions. The results of the discriminant analysis with the three different *a priori* groups also differed from the same analysis conducted with subdivision 26 alone; significant separation of the groups containing the years with high (>30%) M74 incidence from the others (intermediate= 30%>M74>10% and low=M74<10%) was detected only with the abiotic and combined biotic and abiotic variables and only when a one-year lag accounting for migration and feeding was taken into consideration (Table S1-S2; subdivision 26 and subdivisions 25, 26 and 28-2, respectively). However, it is important to note that the misclassification error for the combined data (subdivisions 25, 26 and 28-2) was relatively high in comparison to the corresponding error when focusing on subdivision 26.

Similar to the results for subdivision 26 only, significant canonical correlations were detected for various abiotic and biotic variables in the combined data when compared against the yearly mean M74 incidence (Fig. S3-S4); however, stronger correlations were found when using matching years for the abiotic and combined biotic and abiotic variable datasets than when the one-year lag to account for migration and feeding history was taken into consideration. Some abiotic and biotic variables, such as oxygen and salinity (except during the summer period), phosphate, nitrate, sprat and juvenile herring, had similar moderate to strong correlations with the annual mean M74 incidence, as found for subdivision 26. However, when all three subdivisions were combined and averaged for the analysis, some new correlations were also detected, such as a positive correlation for late autumn and early winter nitrate and nitrate/nitrite, spring Dinophyceae and summer Cryptophyceae and total phytoplankton biomass. Similarly, some correlations were only detected when data from only subdivision 26 were used in the analysis. For nutrients, negative correlations were found between M74 incidence and spring and winter silica concentrations. In terms of biotic variables, positive correlations were found between M74 incidence and summer Diatomophyceae and *Acartia* spp. abundance, and negative correlations were identified between M74 abundance and summer Chrysophyceae and spring *Pseudocalanus* spp. biomass.

**M74 and environmental parameters in the main feeding area in 1991-2013**

Data for cod only covered the period from 1991 to 2013 for the different subdivisions (Fig. S1) and were hence not included in the full analysis. However, when years with cod data were included in the analysis for subdivision 26, a strong canonical correlation was detected both when matching years (t^2^ = 0.80, p = 0.002) and a one-year lag to account for migration and feeding history (t^2^ = .92, p = 0.0006) were considered (Fig. S5). For most of the environmental parameters, the correlations were similar to those obtained from the full analysis excluding cod. For the biotic parameters, some new correlations arose when the cod data were included, such as a positive correlation for the phytoplankton classes Chlorophyceae, Dinophyceae and Prasinophyceae in spring and the total phytoplankton, copepod, *Pseudocalanus* spp., and large herring biomass in summer. Similarly, negative correlations between M74 incidence and Ciliophora biomass in spring and Chrysophyceae biomass in summer were detected only when the presence of cod was considered to illustrate the connections among the different trophic levels. However, in both cases, only small cod (<30 cm) showed a positive correlation with M74 incidence (canonical correlation 0.62 and 0.6, respectively).

**M74 and environmental parameters in the surface versus bottom in the main feeding area**

In the main text, we report results on the basis of whole water column averages. Separate analyses were also performed for the surface water in the upper 10 m layer and bottom waters (i.e. >10 m). Similar to the entire water column, the PCO followed by CAP showed significant separation of the *a priori* groups of the years with high (>30%) M74 incidence from the others (intermediate= 30%>M74>10% and low=M74<10%) when both matching years and a one-year lag to account for migration and feeding history were taken into consideration (Table S3). However, it is important to note that the misclassification error was generally higher in the bottom layer than at the surface and with the one-year lag to account for migration and feeding history than with the matching years. Interestingly, the group of years with high M74 incidence was more obvious among the data from the bottom layer than those from the surface and when data from the entire water column were used in the analysis than when only data from the bottom or surface were used.

Different environmental variables showed stronger correlations in different depth layers, whereas the bottom and entire water column showed similar trends, with nitrogen and oxygen showing a positive correlation with M74 incidence and phosphate and silica having a negative correlation; only nitrate, nitrite + nitrate and TotN and chlorophyll a in the spring showed a positive correlation with M74 incidence in the upper 10 meter layer (Fig. S6).
