# Supplementary figures and images for: Deficiency syndromes in top predators associated with large-scale changes in the Baltic Sea ecosystem
Source: PLoS One. 2020 Jan 9;15(1):e0227714. doi: 10.1371/journal.pone.0227714 (PMC6952091; doi:10.1371/journal.pone.0227714)

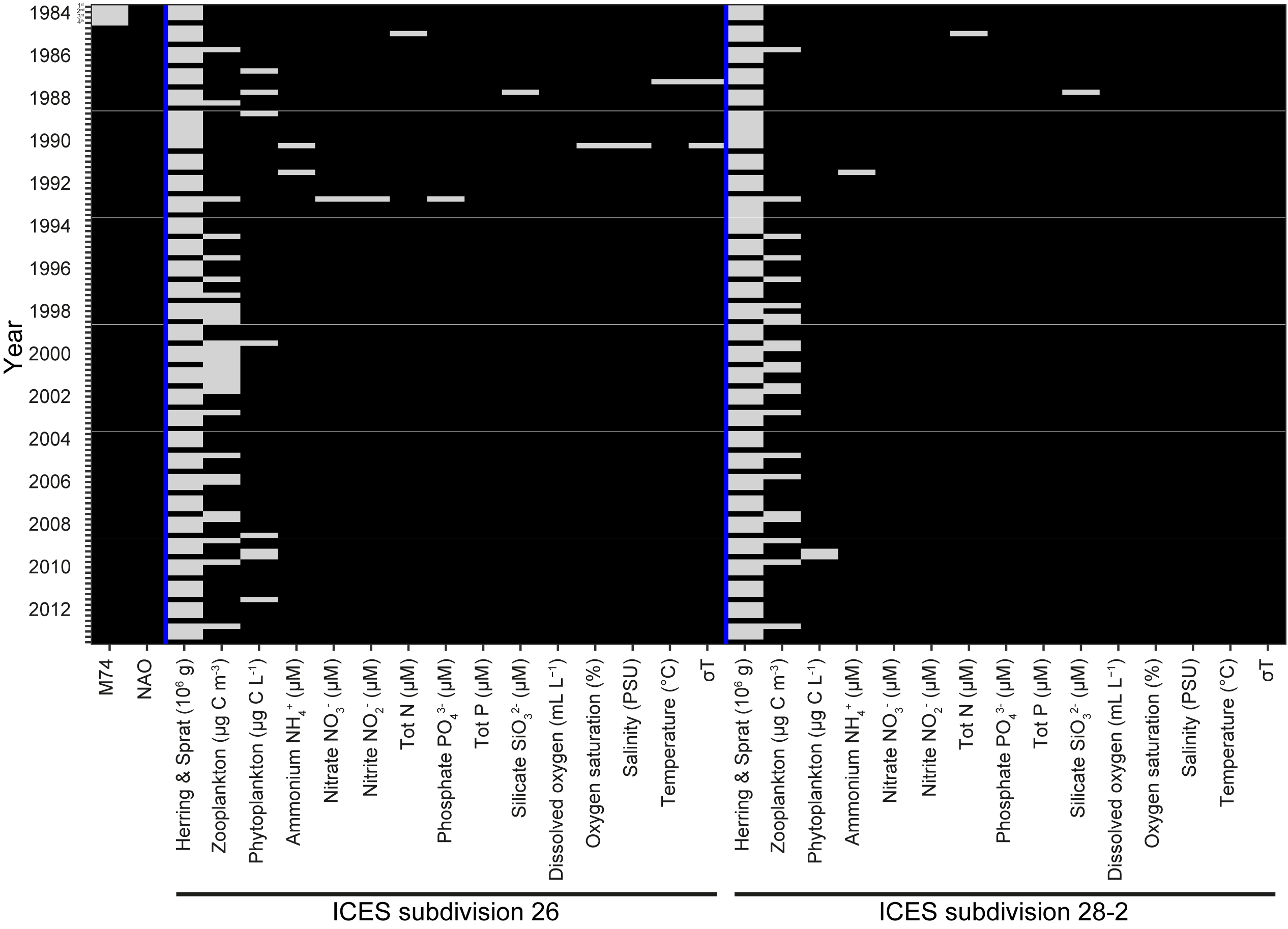

Supplement: S1 Fig — Gray areas indicate missing data, and black areas indicate the presence of data. (TIF) [file pone.0227714.s002.tif]

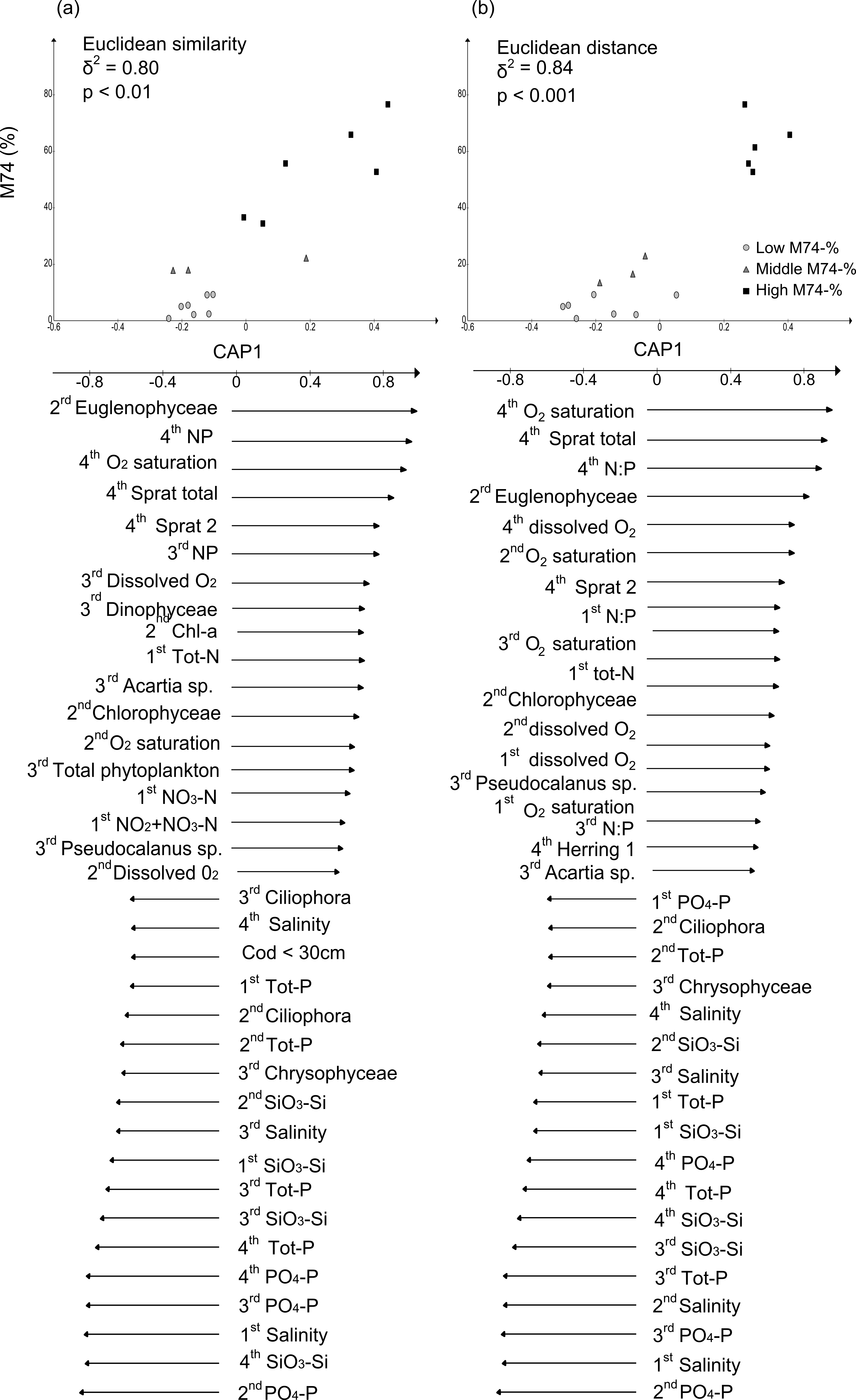

Supplement: S5 Fig — Principal coordinate analysis (PCO) followed by canonical correlation analysis based on distances, (a) same year and (b) 1-year delay (upper panel) including cod in subdivision 26. The intensity of the M74 incidence (high >30%, intermediate 10–30%, and low <10%) is indicated by the shape of the data points. Choice of m with first squared canonical correlation: (a) 5 with 0.8 and (b) 4 with 0.8. Variables with moderate or strong correlations (>0.6) with the canonical axis are listed for all datasets (lower panel, arrows). (PNG) [file pone.0227714.s006.png]

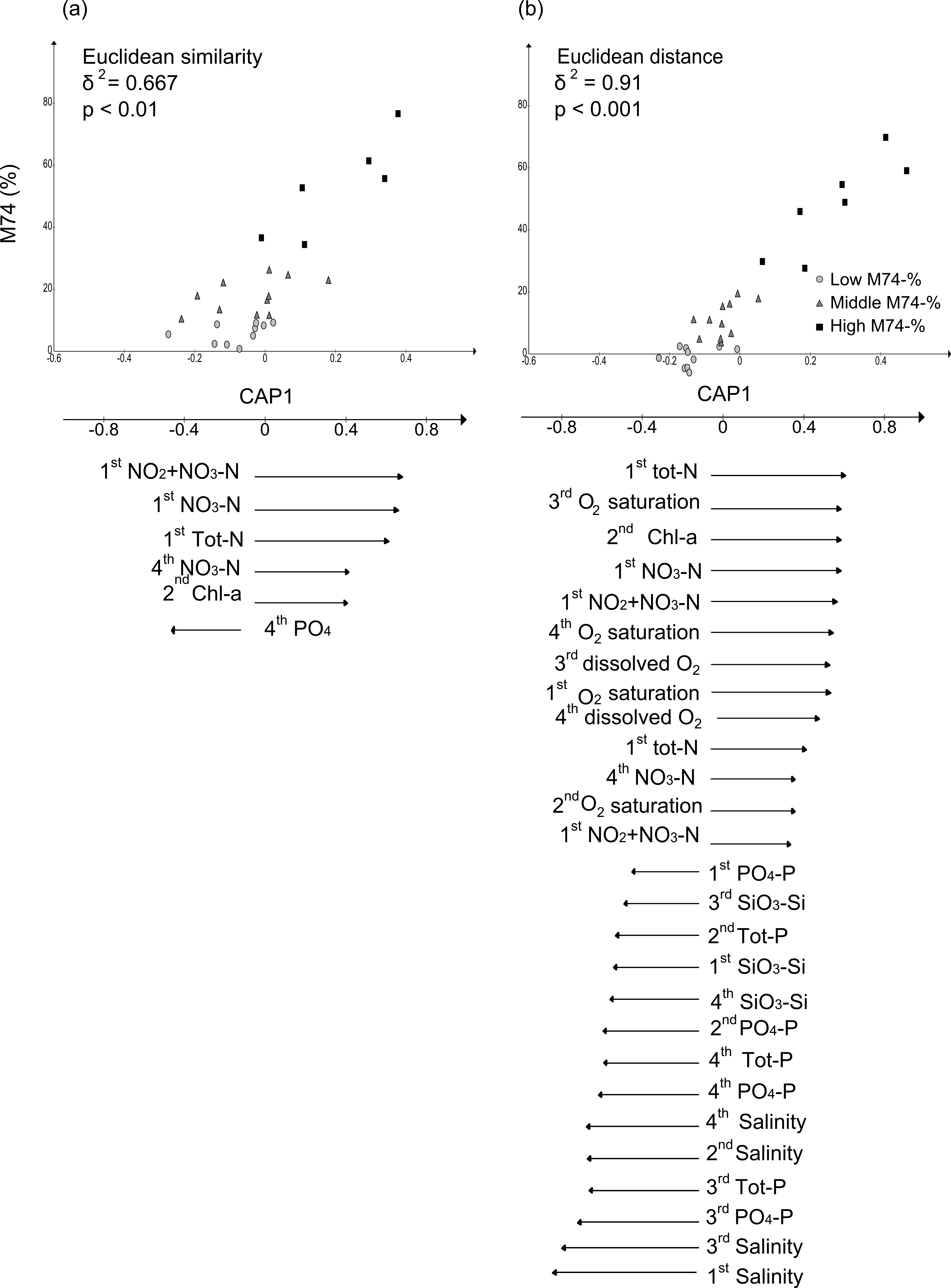

Supplement: S6 Fig — Principal coordinate analysis (PCO) followed by canonical correlation analysis based on distances, (a) upper 10 m layer and (b) bottom layer with 1-year delay (upper panel) in subdivision 26. The intensity of the M74 incidence (high >30%, intermediate 10–30%, and low <10%) is indicated by the shape of the data points. Choice of m with first squared canonical correlation: (a) 8 with 0.7 and (b) 14 with 0.9. Variables with moderate or strong correlations (>0.4) with the canonical axis are listed for all datasets (lower panel, arrows). (PNG) [file pone.0227714.s007.png]

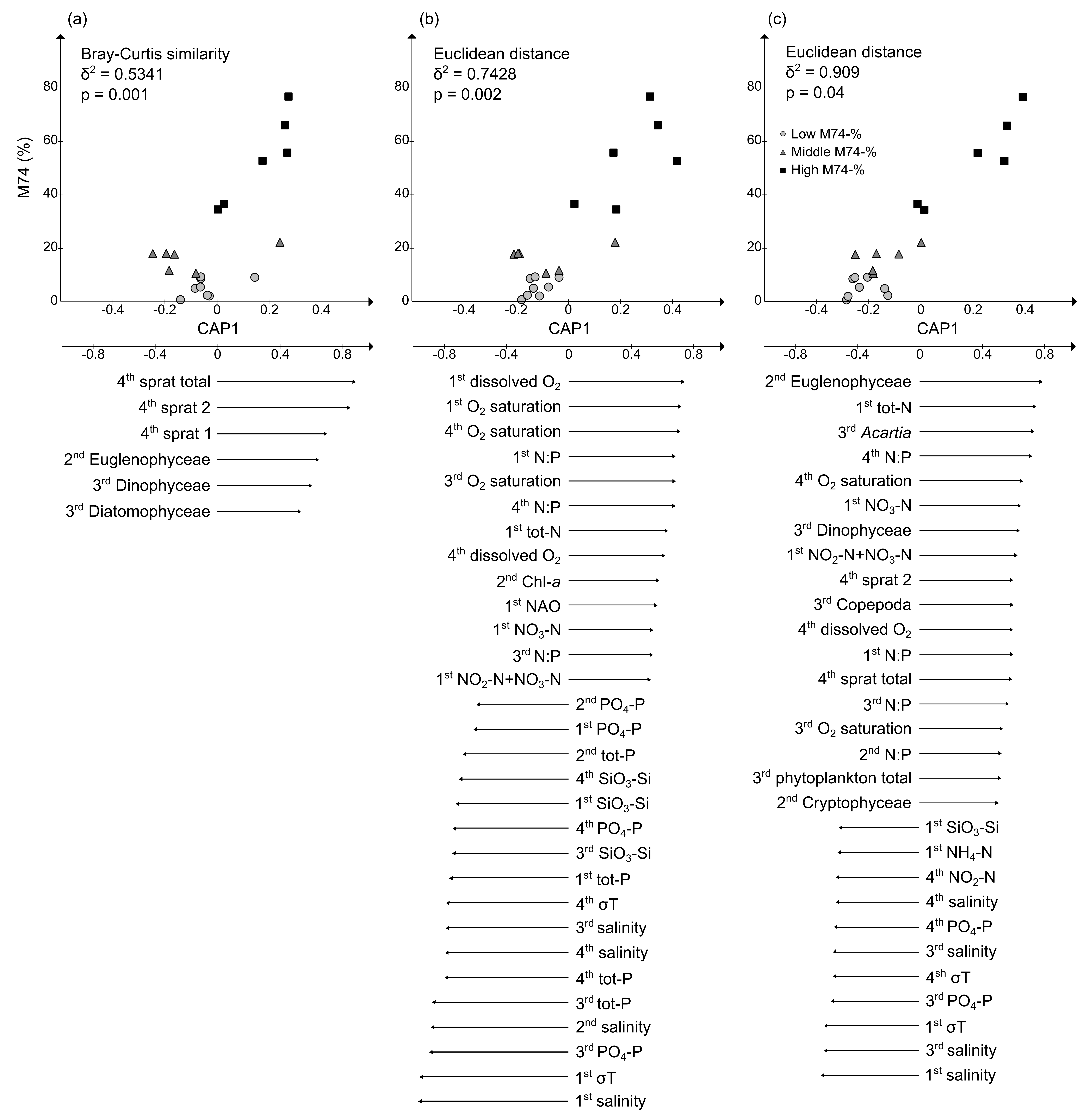

Supplement: S7 Fig — Principal coordinate analysis (PCO) followed by canonical correlation analysis based on distances, (a) biotic, (b) abiotic and (c) both biotic and abiotic variables combined when matching years were used (upper panel) in subdivision 26. The intensity of the M74 incidence (high >30%, intermediate 10–30%, and low <10%) is indicated by the shape of the data points. Choice of m with first squared canonical correlation: (a) 2 with 0.5, (b) 5 with 0.7 and (c) 13 with 0.9. Variables with moderate or strong correlation (>0.4) with the canonical axis are listed for all datasets (lower panel, arrows). (PNG) [file pone.0227714.s008.png]

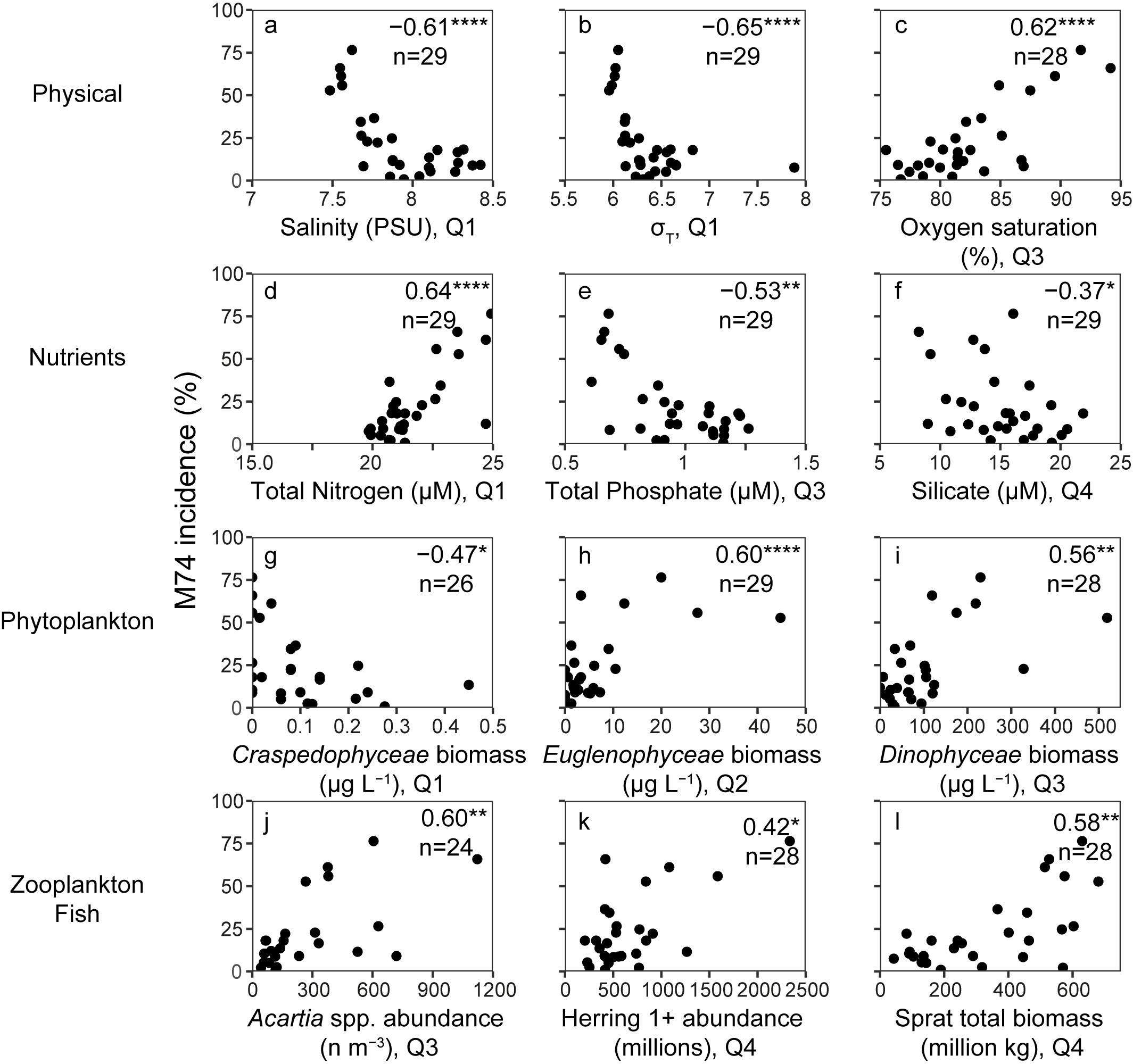

Supplement: S8 Fig — Examples of significant correlations (Spearman rank) between M74 incidence and environmental variables, including salinity (a), σT (b), oxygen saturation (c), total nitrogen (d), total phosphate (e), silicate (f), Craspedophyceae (g), Euglenophyceae (h), Dinophyceae (i), Acartia spp. (j), herring (k) and sprat (l). Spearman´s rank correlation coefficients and number of years in top right corner of each subgraph. Significance level indicated by asterisks, p<0.0001 (****), p<0.001 (***), p<0.01 (**), p<0.05 (*). For all remaining significant correlations, see S4 Table. (TIF) [file pone.0227714.s009.tif]
