# Supplementary material for: Deficiency syndromes in top predators associated with large-scale changes in the Baltic Sea ecosystem
Source: PLoS One. 2020 Jan 9;15(1):e0227714. doi: 10.1371/journal.pone.0227714 (PMC6952091; doi:10.1371/journal.pone.0227714)
Supplement: S2 Fig — Choice of m with first squared canonical correlation: 7 with 0.9. Variables with a correlation >0.7 with the canonical axis are shown. (PPTX) [file pone.0227714.s003.pptx]

## Slide 1
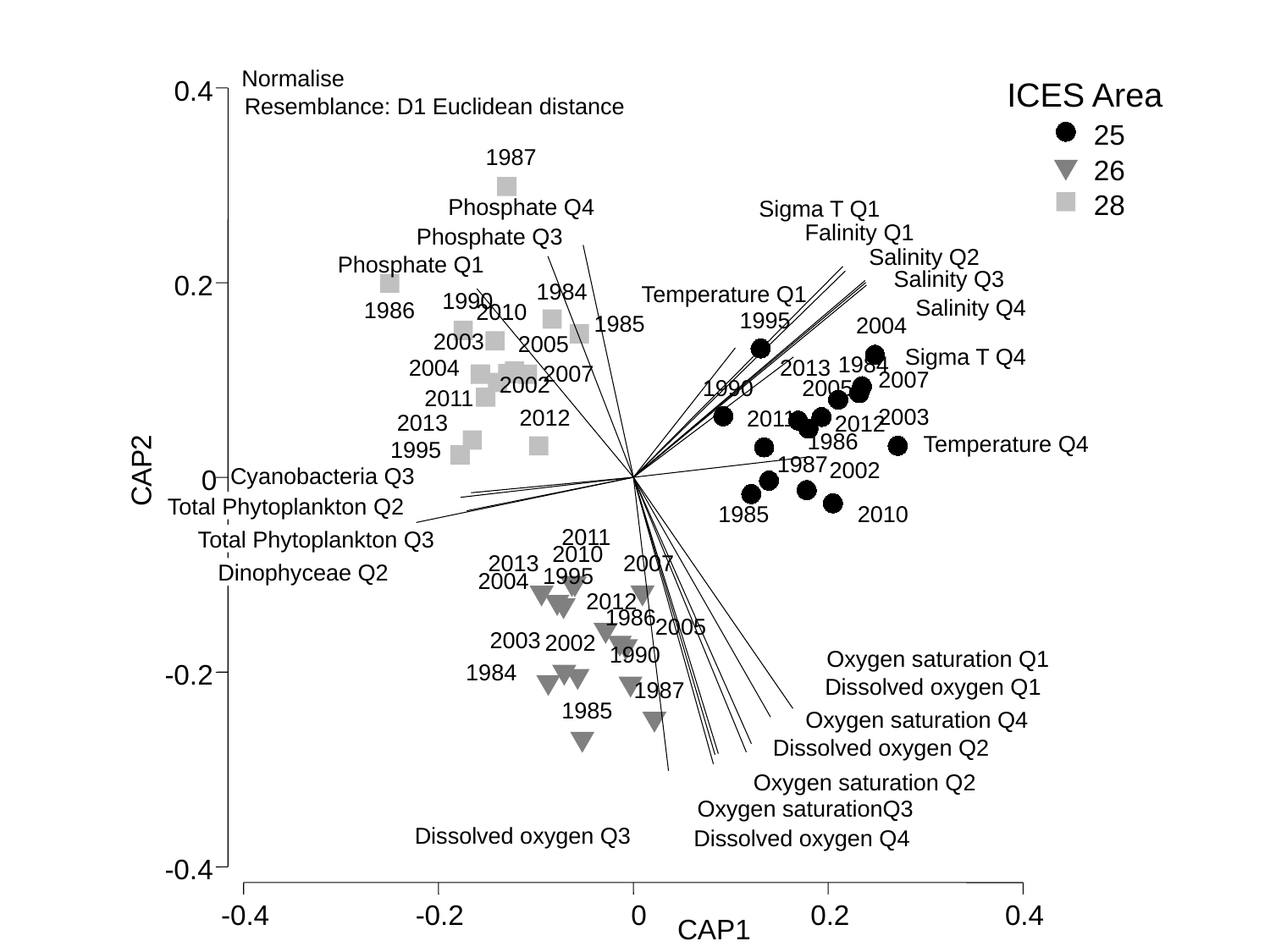

Normalise
0.4
ICES Area
Resemblance: D1 Euclidean distance
25
1987
26
28
Phosphate Q4
Sigma T Q1
Falinity Q1
Phosphate Q3
Salinity Q2
Phosphate Q1
Salinity Q3
0.2
1984
Temperature Q1
1990
Salinity Q4
1986
2010
1995
1985
2004
2003
2005
Sigma T Q4
1984
2013
2004
2007
2007
2002
1990
2005
2011
2003
2012
2011
2013
2012
2
1986
Temperature Q4
1995
P
1987
2002
A
Cyanobacteria Q3
0
C
Total Phytoplankton Q2
1985
2010
2011
Total Phytoplankton Q3
2010
2013
2007
Dinophyceae Q2
1995
2004
2012
1986
2005
2003
2002
1990
Oxygen saturation Q1
-0.2
1984
Dissolved oxygen Q1
1987
1985
Oxygen saturation Q4
Dissolved oxygen Q2
Oxygen saturation Q2
Oxygen saturationQ3
Dissolved oxygen Q3
Dissolved oxygen Q4
-0.4
-0.4
-0.2
0
0.2
0.4
CAP1
