# Supplementary material for: Deficiency syndromes in top predators associated with large-scale changes in the Baltic Sea ecosystem
Source: PLoS One. 2020 Jan 9;15(1):e0227714. doi: 10.1371/journal.pone.0227714 (PMC6952091; doi:10.1371/journal.pone.0227714)
Supplement: S1 Table — The upper light gray panel shows the results for the matching years, and the lower dark gray panel provides the results for when the 1-year delay (salmon migration and feeding) was taken into consideration. (DOCX) [file pone.0227714.s010.docx]

**Table S1.** Results of discriminant analysis with the three different *a priori* groups: high (>30%), intermediate (30%>M74>10%) and low (<10%) M74 incidence with biotic, abiotic and combined biotic and abiotic variable datasets for ICES subdivision 26. The upper light gray panel shows the results for the matching years, and the lower dark gray panel provides the results for when the 1-year delay (salmon migration and feeding) was taken into consideration.

| **Subdivision 26** |  | |  | |  | |  | |  | |  | |  | | |  | |
| --- | --- | --- | --- | --- | --- | --- | --- | --- | --- | --- | --- | --- | --- | --- | --- | --- | --- |
|  | δ^2^_1_ | t_2_ | | p- value | | m | | Misclassification error (%) | | % Correct | | | | |  | | |
|  |  |  |  |  |  |  |  |  |  | High | | Intermediate | | | Low | | |
| Biotic | 0.60 | 0.54 | | 0.05 | | 3 | | 40 | | 67 | | 83 | | 38 | | |  |
| Abiotic | 0.91 | 0.62 | | **<0.05** | | 4 | | 35 | | 67 | | 33 | | 88 | | |  |
| Biotic+Abiotic | 0.97 | 0.68 | | **0.01** | | 5 | | 40 | | 67 | | 33 | | 75 | | |  |
| Biotic | 1.08 | 0.81 | | **<0.05** | | 5 | | 35 | | 100 | | 43 | | 63 | | |  |
| Abiotic | 1.14 | 0.83 | | **<0.001** | | 4 | | 15 | | 100 | | 71 | | 88 | | |  |
| Biotic+Abiotic | 1.23 | 0.85 | | **<0.001** | | 3 | | 15 | | 100 | | 71 | | 88 | | |  |
