# Supplementary material for: Deficiency syndromes in top predators associated with large-scale changes in the Baltic Sea ecosystem
Source: PLoS One. 2020 Jan 9;15(1):e0227714. doi: 10.1371/journal.pone.0227714 (PMC6952091; doi:10.1371/journal.pone.0227714)
Supplement: S2 Table — The upper light gray panel shows the results for the matching years, and the lower dark gray panel provides the results when the 1-year delay (salmon migration and feeding) was taken into consideration. (DOCX) [file pone.0227714.s011.docx]

**Table S2.** Results of discriminant analysis with the three different *a priori* groups: high (>30%), intermediate (30%>M74>10%) and low (<10%) M74 incidence with biotic, abiotic and combined biotic and abiotic variable datasets for ICES subdivisions 25, 26 and 28-2 combined. The upper light gray panel shows the results for the matching years, and the lower dark gray panel provides the results when the 1-year delay (salmon migration and feeding) was taken into consideration.

| **Subdivisions 25+26+28-2** | | | |  | |  | |  |  | | |  | | |  |
| --- | --- | --- | --- | --- | --- | --- | --- | --- | --- | --- | --- | --- | --- | --- | --- |
|  | δ^2^_1_ | t_2_ | p- value | | m | | Misclassification error (%) | | % Correct | | | | | | |
|  |  |  |  |  |  |  |  |  | High | Intermediate | | | | High | |
| Biotic | 0.40 | 0.64 | 0.20 | | 6 | | 60 | | 33 | | 36 | | 50 | | |
| Abiotic | 0.57 | 0.43 | 0.06 | | 4 | | 48 | | 67 | | 27 | | 75 | | |
| Biotic+Abiotic | 0.49 | 0.74 | 0.07 | | 6 | | 40 | | 67 | | 55 | | 63 | | |
| Biotic | 0.22 | 0.23 | 0.53 | | 3 | | 62,5 | | 80 | | 30 | | 22 | | |
| Abiotic | 1.59 | 0.86 | **0.04** | | 15 | | 42 | | 40 | | 70 | | 56 | | |
| Biotic+Abiotic | 1.76 | 0.97 | **0.03** | | 17 | | 50 | | 20 | | 60 | | 56 | | |
