# Supplementary material for: Deficiency syndromes in top predators associated with large-scale changes in the Baltic Sea ecosystem
Source: PLoS One. 2020 Jan 9;15(1):e0227714. doi: 10.1371/journal.pone.0227714 (PMC6952091; doi:10.1371/journal.pone.0227714)
Supplement: S4 Table — The remaining significant correlations between M74 incidence and environmental variables are listed in this table. Significance level indicated by asterisks, p<0.0001 (****), p<0.001 (***), p<0.01 (**), p<0.05 (*). (DOCX) [file pone.0227714.s013.docx]

**Table S4.** Examples of significant correlations (Spearman’s rank) between M74 incidence and environmental variables are shown in Fig. S8. The remaining significant correlations between M74 incidence and environmental variables are listed in this table. Significance level indicated by asterisks, p<0.0001 (****), p<0.001 (***), p<0.01 (**), p<0.05 (*).

|  | **Variable** | **Quarter** | **Spearman's rank correlation coefficient** | ***Significance level*** | **n** |
| --- | --- | --- | --- | --- | --- |
| **Physical** | Salinity (PSU) | 2 | -0.48 | ** | 29 |
|  |  | 3 | -0.56 | ** | 28 |
|  |  | 4 | -0.53 | ** | 29 |
|  | σ_T_ | 2 | -0.40 | * | 29 |
|  |  | 3 | -0.57 | ** | 27 |
|  |  | 4 | -0.56 | ** | 29 |
|  | Oxygen saturation (%) | 1 | 0.46 | * | 29 |
|  |  | 4 | 0.47 | * | 29 |
|  | Dissolved oxygen (mL L^-1^) | 3 | 0.46 | * | 29 |
|  |  | 4 | 0.42 | * | 29 |
| **Nutrients** | Nitrate (μM) | 1 | 0.53 | ** | 28 |
|  |  | 4 | 0.40 | * | 29 |
|  | Nitrite + Nitrate (μM) | 1 | 0.49 | ** | 28 |
|  |  | 4 | 0.38 | * | 29 |
|  | Nitrite (μM) | 4 | -0.47 | * | 29 |
|  | Phosphate (μM) | 2 | -0.39 | * | 29 |
|  |  | 3 | -0.59 | **** | 29 |
|  |  | 4 | -0.59 | **** | 29 |
|  | Total phosphate (μM) | 4 | -0.46 | * | 29 |
| **Phytoplankton** | Chlorophyll *a* (μg L^-1^) | 2 | 0.42 | * | 29 |
|  |  | 3 | 0.66 | ** | 21 |
|  | Chlorophyceae (μg L^-1^) | 1 | 0.55 | ** | 26 |
|  | Chrysophyceae (μg L^-1^) | 1 | -0.64 | **** | 26 |
|  |  | 3 | -0.41 | * | 28 |
|  | Cryptophyceae (μg L^-1^) | 2 | 0.46 | * | 29 |
|  |  | 3 | 0.48 | * | 28 |
|  | Cyanobacteria (μg L^-1^) | 4 | 0.53 | ** | 25 |
|  | Euglenophyceae (μg L^-1^) | 1 | 0.44 | * | 26 |
|  | Prasinophyceae (μg L^-1^) | 2 | 0.40 | * | 29 |
|  |  | 3 | 0.48 | ** | 28 |
| **Zooplankton** | *Acartia* spp. abundance (n m^-3^) | 2 | 0.41 | * | 25 |
|  | *Acartia* spp. biomass (μg m^-3^) | 3 | 0.53 | ** | 24 |
| **Fish** | Sprat 1+ abundance (millions) | 4 | 0.45 | * | 28 |
|  | Sprat 1+ biomass (million kg) |  | 0.48 | * | 28 |
|  | Sprat 2+ abundance (millions) | 4 | 0.46 | * | 28 |
|  | Sprat 2+ biomass (million kg) |  | 0.48 | ** | 28 |
|  | Sprat 2+ abundance (millions) | 4 | 0.51 | ** | 28 |
