# Supplementary material for: Deficiency syndromes in top predators associated with large-scale changes in the Baltic Sea ecosystem
Source: PLoS One. 2020 Jan 9;15(1):e0227714. doi: 10.1371/journal.pone.0227714 (PMC6952091; doi:10.1371/journal.pone.0227714)
Supplement: S5 Table — (DOCX) [file pone.0227714.s014.docx]

**Table S5.** Summary of variables.

| Data type | Parameter | Unit | Source |
| --- | --- | --- | --- |
| Nutrients | Ammonium, NH_4_^+^ | µM | Svenskt HavsARKiv (SHARK) database hosted by the Swedish Meteorological and Hydrological Institute (SMHI). |
|  | Nitrate, NO_3_^-^ |  |  |
|  | Nitrite, NO_2_^-^ |  |  |
|  | Total Nitrogen, TotN |  |  |
|  | Phosphate, PO_4_^3-^ |  |  |
|  | Total Phosphorous, TotP |  |  |
|  | Silica, SiO_3_^2-^ |  |  |
| Hydroclimatic parameters | Temperature | ºC |  |
|  | Salinity | PSU |  |
|  | Dissolved oxygen | mL L^-1^ |  |
|  | Oxygen saturation | % |  |
|  | σT |  |  |
|  | Alkalinity | mmol kg^-1^ |  |
|  | Chlorophyll *a* | µg L^-1^ |  |
|  | pH |  |  |
|  | Secchi depth | m |  |
| Phytoplankton biomass | Chlorophyceae, Chrysophyceae,  Ciliophora, Craspedophyceae, Cryptophyceae, Cyanobacteria, Diatomophyceae, Dinophyceae, Euglenophyceae, Prasinophyceae Prymnesiophyceae | µg C L^-1^ | Baltic Marine Environment Protection Commission - Helsinki Commission (HELCOM) database hosted by ICES |
| Zooplankton biomass | Total zooplankton | µg C m^-3^ | Institute of Food Safety, Animal Health and Environment (BIOR) and Polish National Monitoring Programme |
|  | Total copepods |  |  |
|  | Total cladocerans |  |  |
|  | *Pseudocalanus* spp. |  |  |
|  | *Acartia* spp. |  |  |
|  | *Temora longicornis* |  |  |
| Fish biomass | Herring; age 1, 2+ and total | kg h^-1^ | Baltic International Acoustic Survey [77] |
|  | Sprat; age 1, 2+ and total | kg h^-1^ |  |
|  | Cod; < 30 cm and ≥ 30 cm | kg h^-1^ | Baltic International Trawl Survey [77] |
